# Doxorubicin resistance involves modulation of interferon signaling, transcriptional bursting, and gene co-expression patterns of U-ISGF3-related genes

**DOI:** 10.1016/j.neo.2024.101071

**Published:** 2024-10-13

**Authors:** Pawel Trzaskoma, SeolKyoung Jung, Yuka Kanno, John J. O'Shea, Carson C. Chow

**Affiliations:** a National Institute of Arthritis and Musculoskeletal and Skin Diseases, National Institutes of Health, Bethesda, MD, USA; b National Institute of Diabetes and Digestive and Kidney Diseases, National Institutes of Health, Bethesda, MD, USA

**Keywords:** Chemoresistance, JAK/STAT, Transcriptional bursting, Mathematical modeling, scRNA-Seq, Colorectal cancer

## Abstract

Chemotherapy, although effective in treating cancer, can induce various cellular responses, including senescence and drug resistance. Here, we investigate the transcriptomic alterations induced by doxorubicin (DOX), a commonly used chemotherapeutic agent, in human colon cancer cells. Using single-cell RNA sequencing, we identified distinct cell populations and their transcriptional profiles following subtoxic DOX treatment, revealing cell clusters characterized by differential expression of genes involved in cell cycle regulation and interferon (IFN) signaling. DOX-persisting proliferating cells exhibited upregulation of genes reported to be linked to the unphosphorylated form of ISGF3 (U‐ISGF3) transcription factor. Furthermore, we found that *HSH2D*, a poor prognostic marker, was highly upregulated in doxorubicin-surviving proliferative cells, and its expression was correlated with U-ISGF3-related genes. Analysis of transcription kinetics via mathematical modeling revealed that the number of mRNA molecules produced per transcriptional burst was increased for U-ISGF3-related genes. We also observed altered gene co-expression patterns of U-ISGF3-related genes and others upon DOX treatment, which potentially contributes to chemoresistance of DOX-surviving proliferative cells and may influence cancer cell fate after chemotherapy. Our findings highlight U-ISGF3-related genes and the JAK/STAT pathway as potential therapeutic targets for overcoming chemoresistance in colon cancer.

## Introduction

Since its approval for clinical use in the 1970s, doxorubicin (DOX) has stood out as one of the most potent chemotherapeutic drugs [1]. Unfortunately, DOX chemotherapy has substantial limitations beyond its toxicity; namely, its efficacy can be limited by cancer cells becoming senescent upon treatment [[2], [3], [4], [5], [6]]. Furthermore, much evidence indicates that senescence could increase the risk of metastasis, as therapy-induced senescent cells can reenter the cell cycle [5,[7], [8], [9], [10]]. Moreover, some cancer types are resistant to DOX with clones acquiring resistance during treatment, further increasing the risk of metastasis [11]. Thus, it is important to understand how DOX-surviving proliferative cells differ from senescent cells that respond to the drug or from untreated cancer cells.

It has been shown that the JAK/STAT (the Janus kinase/signal transducer and activator of transcription [12]) pathway can be activated in cancer cells after DOX treatment [13]. For example, transcription factor STAT1 has been shown to be involved in resistance to chemo- and radiotherapy [14,15]. Small cell lung carcinoma (SCLC) cell lines expressing higher levels of STAT1 are more resistant to DOX, the chemotherapy agent etoposide (Etoposide), and radiotherapy [16]. Prolonged low-dose exposure of cells to IFNβ upon chemotherapy-induced DNA damage leads to the formation of the non-canonical protein complex unphosphorylated IFN‐stimulated gene factor 3 (U-ISGF3) [16] that contains unphosphorylated STAT1 and unphosphorylated STAT2 interacting with IRF9. U‐ISGF3 drives the expression of interferon-stimulated genes (ISGs) involved in viral infection and DNA damage that is postulated to increase the resistance to therapy [16]. JAK/STAT inhibition can also sensitize certain resistant cancer types to chemotherapy [17], indicating a possible link between type I interferons (IFN‐I) and chemotherapy resistance.

It remains unclear whether JAK/STAT upregulation upon chemotherapy is mainly a feature of cells that become senescent or cells that do not respond to DOX and continue cycling. It is also not clear how the STAT transcription factors modulate the transcriptional kinetics of ISGs and drive their expression. Finally, it remains to be elucidated whether cancer cells that survive chemotherapy and avoid cell death (DOX-surviving proliferative cells) change their gene co-expression profile at the single-cell level. To date, most studies focus on single selected biomarkers of DOX response or transcriptome changes in bulk, which limits the possibility to answer these questions [18].

In this light, we undertook a single-cell transcriptome-wide approach combined with mathematical modeling to uncover how the transcriptome of DOX-resistant cells is changed in human colon cancer HCT-116 cells. We employed our recently developed StochasticGene software package to infer the potential role of U‐ISGF3 transcription factor in the regulation of transcriptional kinetics and bursting [19]. We found that the modulation of bursting correlates with substantial changes in the co-expression patterns of genes linked to the U-ISGF3 complex, senescence, proliferation, or mitosis. Additionally, DOX-persisting proliferative cells marked by a U‐ISGF3-gene signature upregulate the *HSH2D* gene – a poor prognostic marker in colorectal cancer (Human Protein Atlas proteinatlas.org [20]).

## Results

### Doxorubicin induces IFN target genes in human colon cancer cells

**Fig. 1:**
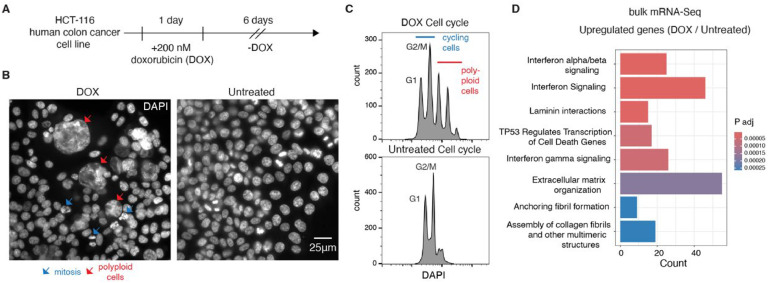
Doxorubicin treatment upregulates IFN-related genes. (A) A scheme of experimental design: human colon cancer cells (HCT-116) were treated with 200 nM doxorubicin (DOX) for 24 hours. After washing out the drug, the cells were analyzed after 6 days. (B) Two representative images of DOX-treated (left) and untreated cells (right). Red arrows indicate polyploid giant cells, and blue arrows indicate mitosis in the DOX-treated sample. The white bar indicates a scale: 25 µm. (C) Flow cytometry panel of fixed DAPI-stained DOX-treated (top) and untreated (bottom) cells reveals subpopulations of cycling (in blue) and polyploid (in red) cells upon DOX treatment. (D) Gene ontology analysis of upregulated genes upon DOX treatment as revealed by bulk mRNA-Seq.

Depending on the dose, DOX can lead to cell death or cell cycle arrest along with senescence. A growing body of evidence indicates that the latter is undesirable, as chemotherapy-induced senescence can be reversible, thereby increasing the risk of metastasis [5,[20], [21], [22]]. At the same time, some cells can develop resistance to DOX and continue cycling after treatment [17]. Our first goal was to uncover distinctions in the transcriptomes of chemo-resistant (DOX-surviving cycling cells), senescent, and untreated human colon cancer cells (Fig. 1A). After treatment, we observed the presence of distinct cell populations consisting of normal-sized dividing cells in either G1 or G2/M stage of the cell cycle (Figs. 1B-C and S1), and polyploid, giant cells characterized by high activity of β-galactosidase, a marker of senescence (Figs. 1B-C and S1).

To uncover changes at the transcriptional level, we performed bulk mRNA-Seq and identified 1864 downregulated and 1875 upregulated genes upon DOX treatment (Fig. S2A, Table S1). Among downregulated genes, we observed enrichment of genes involved in mitosis and cell cycle checkpoints, in line with the presence of a subpopulation of senescent cells (Fig. S2B). On the other hand, among upregulated genes, we identified a strong enrichment of genes involved in IFN signaling, including key genes such as *STAT1, STAT2*, and *IRF9*, which are critical components of the signaling pathway activated by IFN‐I (Fig. 1D, Table S2).

### scRNA-Seq reveals populations of senescent and persisting proliferative cells upon doxorubicin treatment

**Fig. 2:**
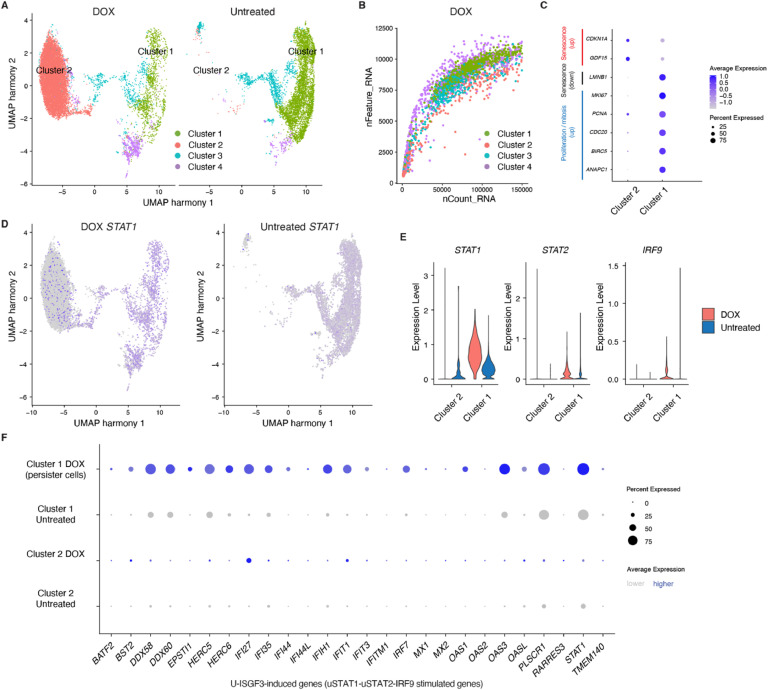
DOX-surviving proliferative cells, but not senescent cells, exhibit upregulated *STAT1* and U-ISGF3-related genes. (A) scRNA-Seq UMAP (Uniform Manifold Approximation and Projection) of DOX-treated (left) and untreated (right) HCT-116 cells. (B) Plot presenting nFeature_RNA (the number of genes detected per cell) vs. nCount_RNA (the number of Unique Molecular Identifiers per cell). (C) Dot plot presenting average expression of marker genes of senescence (in red), *LMNB1*, a gene typically downregulated in senescent cells, and genes involved in proliferation or mitosis (in blue) from combined all samples. (D) *STAT1* expression in DOX-treated (left) and untreated (right) cells. (E) Violin plots presenting expression of *STAT1, STAT2*, and *IRF9* genes in DOX-treated (in red) and untreated cells (in blue). The expression within clusters 2 and 1 is shown. (F) Dot plot presenting average expression of 26 U-ISGF3 (complex formed by unphosphorylated *STAT1*, unphosphorylated *STAT2*, and *IRF9*)-induced genes [16]: U-ISGF3-related genes, detected with scRNA-Seq, within clusters 1 and 2 of untreated and DOX-treated cells.

Although bulk mRNA-Seq revealed almost four thousand significantly affected genes, it was unknown whether their expression was altered in senescent cells or cycling cells that survived DOX treatment. To address this ambiguity, we performed single-cell RNA sequencing (scRNA-Seq), followed by a cluster analysis. We identified four clusters present in both untreated and DOX-treated samples. Since clusters 3 and 4 consisted of too few cells to perform a meaningful analysis, we focused our analyses on the main clusters: cluster 1, which was present in untreated and DOX-treated samples, and cluster 2, which was highly enriched in the DOX sample (Fig. 2A). The major cluster 2 in the DOX sample exhibited a substantially lower number of genes detected per cell (fewer than 1,000 for most cells) compared to cluster 1 (7,000 — 11,000 for most cells) (Fig. 2B), indicating decreased gene activity and lower cellular complexity in DOX induced cluster 2. To characterize both clusters, we examined markers of cell-cycle arrest and proliferation. We found that cells within cluster 2 exhibited higher expression of cell cycle arrest and senescence markers: *CDKN1A*, encoding the cell cycle inhibitor p21 [23], and *GDF15*, encoding growth differentiation factor 15 [23]. Most cells within cluster 2 also lacked expression of *LMNB1*, encoding lamin B1. As loss of lamin B1 is a biomarker of senescence [24], this suggests that cluster 2, enriched in the DOX sample, contains senescent and possibly other aberrant cells (Figs. 2C and S3). On the other hand, cluster 1 contained cells enriched for expression of genes involved in proliferation: *MKI67*, encoding the classical marker of proliferating cells Ki-67, and *PCNA*, encoding the cofactor of DNA polymerase delta, playing a role in genome replication and repair [25]. Moreover, these cells expressed the *CDC20* gene, encoding cell division cycle protein 20 homolog, which can function as an oncoprotein, and *BIRC5*, which inhibits apoptosis; both promote cancer progression, and are poor prognostic markers in cancer patients [26,27]. Finally, cluster 1 cells expressed higher levels of genes facilitating mitosis, such as *ANAPC1*, encoding a subunit of the anaphase-promoting complex (APC) [28]. Altogether, single-cell data suggest that cluster 1 contains dividing, proliferative cells that survived DOX treatment, in contrast to cells within cluster 2 that shows signature of cell cycle arrest and senescence.

### JAK/STAT genes are silenced in a cluster enriched with senescent cells

Since our bulk mRNA-Seq revealed upregulation of genes involved in IFN signaling upon DOX treatment and we subsequently resolved the biological characteristics of clusters 1 and 2 at the single cell level, we further investigated which cluster contained cells upregulating IFN signature genes. It has been shown that IFN‐I can enforce cell senescence [29]. Specifically, IFN‐I activates JAK/STAT pathway genes, and the JAK/STAT pathway has been shown to play a role in replicative senescence [30] and senescent cells in aged mice [31,32]. However, it remains unclear whether this pathway is activated in therapy-induced senescence upon DOX treatment. The JAK/STAT pathway consists of four kinases: JAK1, JAK2, JAK3, and TYK2, and seven transcription factors: STAT1, STAT2, STAT3, STAT4, STAT5A, STAT5B, and STAT6 [33]. In our scRNA-Seq data, JAK/STAT genes were not expressed at detectable levels in the majority of cluster 2 cells (associated with senescence) after DOX treatment (Figs. 2D and S4). However, we note that scRNA-Seq, because of dropouts [19], has lower sensitivity in detecting lowly expressed genes compared to bulk mRNA-Seq.

### DOX-surviving proliferative cells upregulate *STAT1* and genes induced by U-ISGF3

Previously, it has been demonstrated that cell lines of SCLC with high expression of *STAT1, STAT2*, and *IRF9* exhibit increased resistance to DNA damage induced by chemo- and radiotherapy [16]. In canonical IFN signaling, phosphorylated STAT1 and STAT2, along with IRF9, form a transcription factor complex ISGF3, which binds to the promoter of genes involved in the anti-viral response [34]. Although ISGF3 drives the initial response to type I IFNs, the unphosphorylated form of this complex (U‐ISGF3) drives a prolonged response to IFN‐I [16]. Consequently, U‐ISGF3-induced genes contribute to cell resistance to DNA damage [16], as they overlap with the subset of IFN-related DNA damage resistance signature (IRDS) genes [35].

In contrast to cluster 2 cells, cluster 1 DOX persisting proliferative cells expressed the most JAK/STAT genes except for *JAK3* and *STAT4*, as their expression is mainly restricted to immune cell types [33,36] (Figs. 2D-E and S4). *STAT1* was among the predominantly upregulated genes in this pathway upon DOX treatment. Additionally, we observed upregulation of *STAT2* and *IRF9* with DOX in persisting proliferative cells whereas most JAK kinases or other STATs remained stable or showed only subtle changes compared to *STAT1* (Figs. 2E and S4). This suggests an enhanced formation of U-ISGF3 and/or ISGF3 complex in DOX-treated colon cancer cells, similar to SCLC cells [16]. To confirm, we examined a previously identified set of 29 U‐ISGF3-inducible genes by Cheon et al. [16]. Using scRNA-Seq, we found an upregulation of most U‐ISGF3-inducible genes (n= 26 genes; no expression for 3 genes: *BIRC4BP, G1P2, RTP4*) along with *STAT1* upregulation (Fig. 2F). Among the upregulated genes, we identified the *OAS1* gene encoding 2′,5′‐oligoadenylate synthetase 1, which suppresses PARylation, a posttranslational modification that, in response to DNA damage, promotes cell death [37] (Fig. 2F). Additionally, among the upregulated genes, we identified genes that have increased expression in metastatic cancer cells, such as the *BST2* gene [38], or promote cancer cell migration, tumor growth, and angiogenesis like *IFI27* [39,40].

Our scRNA-Seq results show that DOX-surviving proliferative cells have upregulated genes that block cell death and promote metastasis, and these genes are related to the U‐ISGF3 complex [16]. All 3 components of the U-ISGF3 complex are also upregulated in DOX-surviving proliferative cells.

### DOX-resistant proliferative cells remodel co-expression patterns of U-ISGF3-related genes

**Fig. 3:**
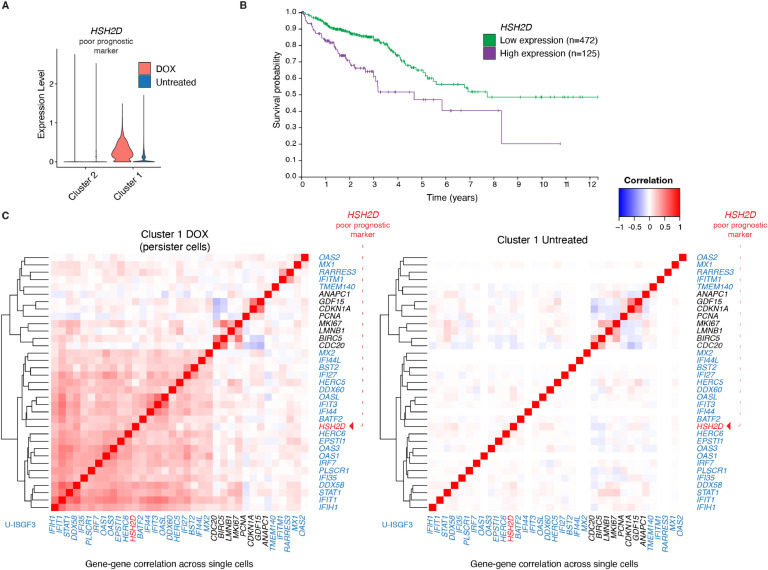
DOX-surviving proliferative cells upregulate the *HSH2D* gene associated with poor survival prognosis. (A) Violin plot showing the upregulation of the *HSH2D* gene in cluster 1 after DOX treatment. (B) Survival probability (in years) for colorectal cancer patients with low and high expression of the *HSH2D* gene - a marker for poor prognosis in colorectal cancer. Credit: modified from Human Protein Atlas proteinatlas.org [20]; available from v23.proteinatlas.org: https://www.proteinatlas.org/ENSG00000196684-HSH2D/pathology/colorectal+cancer (C) Gene-gene co-expression *Pearson* correlation matrices in persister cells (DOX-surviving proliferative cells; cluster 1; left) and untreated cells (cluster 1; right). Hierarchical clustering was performed on the Cluster 1 DOX sample, and the same gene order was applied to the Cluster 1 Untreated sample. *HSH2D* is marked in red. U-ISGF3-related genes are presented in blue (n= 26 genes detected with scRNA-Seq), along with the *LMNB1* gene, typically downregulated in senescent cells, and genes involved in proliferation or mitosis (in black). The red dashed arrows indicate *HSH2D* gene (a poor prognostic marker in colorectal cancer), which clusters together with most U-ISGF3-related genes.

To investigate the consequences of chemoresistance potentially associated with U-ISGF3, we aimed to assess the risk that DOX persisting cells carry for patients’ outcome. To do this, we searched The Human Protein Atlas [20] for poor prognostic markers in colorectal cancer. We identified one gene (*HSH2D*: Hematopoietic SH2 Domain Containing gene) out of the top six prognostic markers for poor outcome that was highly upregulated upon DOX treatment in our data (Fig. 3A). Public data (Human Protein Atlas proteinatlas.org [20]) showed that high expression of *HSH2D* correlates with a significantly lower 5-year survival rate in colorectal cancer patients (65 % for patients with low expression of *HSH2D* vs. 47 % for high expression; P= 9.5e-6) (Fig. 3B). Although relatively little is known about its function, *HSH2D* has been shown to be induced by type I IFNs [41]. Hence, based on the literature and our scRNA-Seq results (Fig. 3A), we hypothesized that *HSH2D* expression might correlate with U-ISGF3-target genes in DOX-surviving proliferative cells.

The preceding analyses were focused on single genes in isolation. Therefore, we took advantage of our scRNA-Seq data to investigate gene co-expression patterns among multiple genes. We pre-selected U-ISGF3-related genes detected by our scRNA-Seq, along with markers of senescence, proliferation, and mitosis (listed in Fig. 2C). The correlation of co-expression of these genes was then calculated across single cells in cluster 1, comparing DOX-treated (DOX-surviving proliferative cells) and untreated conditions (Fig. 3C, Tables S3-4). Correlation equal to 1 means that two genes are expressed together across the cells, whereas anticorrelation (-1) means that if one gene is expressed in the cell, a second one is not. In DOX-surviving proliferative cells, we found positively correlated gene pairs, for example, two senescent markers (*GDF15* and *CDKN1A* R= 0.54) and anticorrelated gene pairs, for example, *GDF15* and genes involved in cancer progression: *CDC20* (R= -0.26) and *BIRC5* (R= -0.17). We also observed that *BIRC5* expression positively correlated with the proliferation marker gene MKI67 (R= 0.52), while the correlation between CDC20 and MKI67 was weaker (R= 0.24) (Fig. 3C, Table S3).

Having observed the expected relation between the analyzed genes, we next performed clustering and found that most U-ISGF3-related genes clustered together in the DOX-treated condition. Importantly, we found that in DOX treated cells, *HSH2D* clustered together with U-ISGF3-related genes and its expression correlated with many U-ISGF3 target genes like *DDX58* (R= 0.37) and *IFIT1* (R= 0.31), or *STAT1* (R= 0.26). Although we note that the correlations were moderate or weak, we observed a substantial increase in correlation in DOX-surviving proliferative cells compared to untreated cells (Fig. 3C). Interestingly, *HSH2D* expression did not correlate with other genes associated with colorectal cancer metastasis and tumorigenesis, namely *CDC20* and *BIRC5* (Fig. 3C, Table S3). In contrast to *HSH2D, CDC20* and *BIRC5* did not cluster together with the majority of U-ISGF3 target genes. For example, we observed almost no correlation between *CDC20* and *STAT1* (R= 0.06), suggesting that high expression of the poor-outcome prognostic marker *HSH2D* could indicate a phenotype of chemoresistance.

In contrast, DOX-untreated cancer cells showed no notable “blocks” of co-expression patterns for the analyzed genes, despite the presence of correlation between single marker genes such as the senescence markers *GDF15* and *CDKN1A* (Fig. 3C, Table S4). *HSH2D* expression also did not correlate with *STAT1* or other U-ISGF3-related genes (Fig. 3C) in untreated cells.

Altogether, using U-ISGF3-related genes as a reference, we identified possible remodeling of the transcriptome in DOX-surviving proliferating cells, where U-ISGF3-related genes were coordinately expressed more tightly together among themselves as well as with *HSH2D*, the unfavorable prognostic marker for colorectal cancer. Our data also show that the outcome of chemotherapy on cancer cells does not solely depend on which genes are expressed, but rather is more dependent on the gene co-expression patterns among them.

### Genes related to U‐ISGF3 are transcribed with larger burst sizes in DOX-surviving proliferative cells

One key aspect of gene regulation is transcriptional bursting, a phenomenon of discontinuous transcription across all genes [42], which has been demonstrated to be targetable by drugs [43]. Bursting can be modulated by either change to the bursting frequency, the burst size (number of mRNAs produced per burst), or both [19,44,45]. It has been speculated that perturbing RNA polymerase II release, which can decrease the bursting frequency, can alter gene networks [19]. Thus, changed co-expression patterns of U-ISGF3-related genes might imply a change in transcriptional bursting in chemo-resistant proliferative cells.

**Fig. 4:**
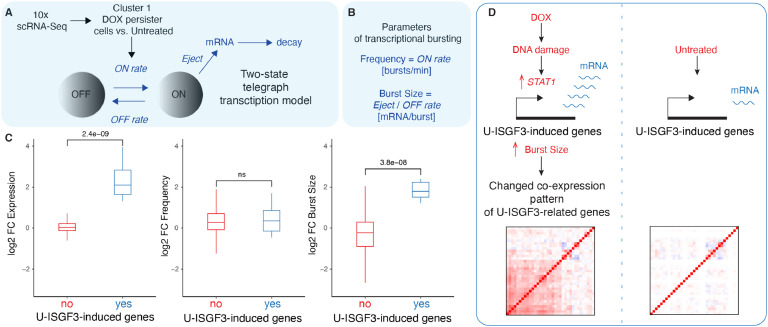
DOX-surviving proliferative cells upregulate U-ISGF3-related genes through increase of transcriptional burst size. (A) Analysis scheme: scRNA-Seq was performed on untreated and DOX-treated HCT-116 cells. scRNA-Seq histograms of mRNA per cell were obtained from cells within cluster 1, and the two-state telegraph transcription model was fitted to histograms for each gene. The model scheme is depicted in a blue box, comprising transitions between an inactive and an active state, from which mRNA is emitted. The model has four parameters: the ON rate (frequency), OFF rate, eject rate (mRNA creation rate), and decay rate reflecting the mRNA disappearance rate [19]. (B) Parameters used to infer bursting kinetics of genes: frequency (ON rate) and burst size, calculated as Eject/OFF rate, corresponding to the mean number of mRNA produced while in the active state. (C) Box plots presenting the log2 fold changes (DOX/Untreated) of expression, frequency and burst size in cells from cluster 1; in blue: U-ISGF3-induced genes [16], in red: all other genes. P-values are shown from the Wilcoxon test, n (yes)= 12 genes that passed quality control (QC) filtering based on ON rate and burst size estimation uncertainty: *DDX58, DDX60, EPSTI1, HERC5, HERC6, IFI27, IFI35, IFIH1, IRF7, OASL, PLSCR1, STAT1*, and n (no; all other genes)= 6363 genes that passed QC filtering. (D) A model of DOX resistance: Doxorubicin induces DNA damage and upregulates genes involved in DNA damage resistance, such as *STAT1* and U-ISGF3-induced genes [16], through an increase in their burst size. As a result, DOX-surviving proliferative cells rearrange the co-expression pattern of U-ISGF3-related genes.

To investigate the potential role of U-ISGF3 in transcriptional bursting, we employed the StochasticGene software package to infer the genome-wide kinetics of transcription [19]. We utilized our scRNA-Seq data and selected cells from cluster 1 from both untreated and DOX-treated samples. Subsequently, we fit the classic two-state stochastic telegraph model to infer the parameters of transcriptional bursting. This model assumes two gene states: inactive and active, from which mRNA is produced and then decays (Fig. 4A). By fitting the model to mRNA histograms obtained with scRNA-Seq, we can infer the ON and OFF rates, which represent the transition rates between gene states, as well as the eject rate, which is the mRNA creation rate. The ON rate provides insights into the frequency of bursting (bursts per minute), indicating how often genes are turned on, while the OFF and eject rates are used to calculate burst size (eject/OFF), representing the number of mRNA molecules produced per burst (Fig. 4B). Since ON rate and burst size correlate the best with imaging-based single molecule RNA FISH [19], we focused our analysis on these two parameters (Fig. 4B). Additionally, we performed quality control of inferred rates based on ON rate and burst size estimation uncertainty and analyzed only those genes that passed this control.

Our analysis revealed that U‐ISGF3-related genes [16] upregulated in DOX-surviving proliferative cells that passed quality control exhibit significantly larger burst sizes compared to other genes (P= 3.8e-08) (Fig. 4C, Table S5). While the bursting frequency between these two groups did not show significant differences, we observed a slight increase in the frequency of bursting genome-wide for both U‐ISGF3-related and all other genes across the transcriptome (Figs. 4C, Table S5). Based on our microscopic, flow cytometry, and scRNA-Seq analyses, we infer that the majority of proliferating cluster 1 cells are diploid. To ensure that the potential presence of polyploid cells within cluster 1 does not impact our results regarding U‐ISGF3-related genes exhibiting larger burst sizes, we conducted additional controls. Assuming that DOX-treated cluster 1 cells have 4, 8, and 16 copies of each chromosome, we refit our model (Fig. S5A). Consequently, we observed significantly larger burst sizes of U‐ISGF3-related genes (Fig. S5B-D). As expected, altering the assumption of the number of alleles primarily affected the overall frequencies of bursting for all genes. However, we observed no significant difference between U-ISGF3-related genes and other genes in this regard (Fig. S5B-D).

Based on the collected evidence and published data, we propose a model of DOX-resistance in which drug treatment induces DNA damage and upregulates *STAT1* and other genes involved in anti-viral and DNA damage response pathways. U‐ISGF3-related genes [16] in DOX-surviving proliferative cells are expressed with larger burst sizes, thereby increasing their mean expression and altering their co-expression pattern. Among these genes, in agreement with previous studies [16], we have identified ones that can inhibit cell death and promote tumor progression and metastasis (Fig. 4D).

In summary, our analyses collectively provide mechanical insights into the potential role of U‐ISGF3 in remodeling transcription that could not be inferred by analyzing the transcriptome at bulk mRNA levels. Our single-cell approach allowed understanding of not only the global gene expression landscape but also the dynamics of transcript production and how the U-ISGF3 signature represents the phenotype of DOX-surviving proliferative cells.

## Discussion

The recent development of single-cell methods and analytical modeling that can be applied to the entire genome has allowed us to examine the broad impact of DOX rather than looking at selected genes in isolation. In this context, we applied a single-cell transcriptome-wide approach combined with mathematical modeling to uncover how the transcriptome of DOX-resistant cells is altered. Our results suggest the potential involvement of the U-ISGF3 transcription factor complex in modulating transcriptional bursting of its target genes in DOX-surviving proliferative cells, as well as changes to the gene co-expression profile upon DOX treatment.

### Doxorubicin resistance and the role of JAK/STAT pathway

As a model of chemotherapy resistance, we chose DOX treatment at a dose that induces a heterogeneous response in colon cancer cells, leading to the emergence of distinct cell populations: senescent cells and DOX-surviving proliferative cells. We found that the latter exhibit upregulation of *STAT1* and other components of the U-ISGF3 complex (*STAT2* and *IRF9*), as well as genes reported to be induced by this complex [16]. This suggests that the U-ISGF3 complex may play an important regulatory role in the survival and proliferation of DOX-resistant cells. This agrees with previous studies in SCLC, where the JAK/STAT pathway has been shown to be involved in DNA damage resistance upon chemo- and radiotherapy through activation of U-ISGF3-inducible genes [16]. Moreover, cell lines expressing higher levels of STAT1, STAT2, and IRF9 have been shown to be more resistant to DNA damage. The same study also revealed that silencing of STAT1 in fibroblasts decreases cell viability after DOX treatment [16]. The U-ISGF3 complex, known for its role in prolonged IFN response and DNA damage resistance, drives the expression of genes that inhibit cell death and promote metastasis [16]. There is also an overlap of the U-ISGF3-inducible genes with genes classified as the IFN-related DNA damage resistance signature (IRDS) [37,46,47], such as *OAS1*, which prevents cell death after DNA damage [37].

Besides *STAT1* and *STAT2*, other components of the JAK/STAT pathway have been studied in the pathogenesis of cancer, including *STAT3* [48]. JAK/STAT genes have also been studied in senescence. For example, the JAK/STAT pathway has been shown to be activated in aged tissues [31,32] and during replicative senescence [49]. JAK/STAT signaling has also been shown to be activated in drug-induced senescence [50]. Our bulk mRNA-Seq showed significant upregulation of this pathway upon DOX treatment and scRNA-Seq located the JAK/STAT signature to the cluster of DOX-surviving proliferative cells. The expression of JAK/STAT genes other than the component of the ISGF3 complex was less affected by DOX treatment. Altogether, our results, in line with previous reports in other cancer cell types, suggest a potential role of the U-ISGF3 transcription factor in supporting resistance to DOX.

### Transcriptional bursting and gene co-expression patterns altered by doxorubicin

Transcriptional bursting has been extensively studied as a key aspect of gene regulation [42], and also in the context of the development of therapy resistance [51]. Two of the most commonly analyzed features of this process are bursting frequency and burst size, which inform how often genes are turned on and, if so, how many mRNA molecules they produce in a single burst. Modulation of these parameters can either decrease or increase transcription. Hence, it is important to understand how this process is regulated. A better understanding can contribute to the development of potential future drugs aimed at reducing the risk of chemoresistance. Although most studies of bursting employ microscopic approaches to study the dynamics of selected genes, recently we and others [19,52,53] have employed scRNA-Seq and mathematical modeling to infer transcriptional dynamics transcriptome-wide. Using this approach, we showed that enhancers and Mediator complex subunit MED26 regulate the frequency of bursting, knockout of the *Myc* oncogene impacts burst size, while other treatments including cohesin loss and BRD4 perturbation can affect both [19]. Here, we applied our tool to investigate the mechanism behind altered transcriptional dynamics of U-ISGF3-related genes in DOX-treated cells. We observed that these genes exhibit larger transcriptional burst sizes, indicating a higher number of mRNA molecules produced per transcriptional burst. This increase in burst size, rather than frequency, contributes to the enhanced expression of chemoresistance-related genes. Moreover, our analysis reveals substantial changes in the gene co-expression patterns in DOX-surviving proliferative cells. We show that DOX-resistant cells, in contrast to untreated cells, exhibit a highly coordinated, enhanced expression of U-ISGF3-related genes. It is important that the co-expression analysis was conducted independently of the bursting analysis. We speculate that there may be a connection between these two phenomena. Our results suggest that bursting frequency [19,54] and larger burst sizes can increase gene-gene co-expression by producing more mRNA simultaneously. Increased burst size could enhance the correlated expression of genes that are co-regulated or functionally related, such as U-ISGF3-related genes. It would also be interesting to know whether transcriptional bursting plays a similar role in cluster 2 of DOX-treated cells which exhibited low gene expression and reduced complexity. However, this investigation presents significant technical challenges due to several factors. Low and heterogenic expression within this cluster resulting in bimodal mRNA per cell histograms for some genes, and uncertainty regarding possible polyploidy and number of alleles all contribute to these difficulties. These factors make model fitting challenging for producing high-confidence output. Instead, further investigation will require microscopic approaches that allow for detection of even lowly expressed genes and accurate identification of polyploid cells.

### Doxorubicin resistance can lead to poor outcomes in patients

To further investigate the gene co-expression profile of DOX-resistant cells, we searched the literature for poor prognostic markers. Among the top six markers of unfavorable prognosis in colorectal cancer (The Human Protein Atlas [20]), we found one highly upregulated gene in the cluster of DOX-surviving proliferative cells: the *HSH2D* gene, whose higher expression correlates with lower survival rates (The Human Protein Atlas [20]). Our gene co-expression analysis revealed that *HSH2D* expression correlates with the expression of many U-ISGF3-related genes in DOX-surviving proliferative cells. In line with our findings, *HSH2D* has been reported as a type I IFN-stimulated gene [41]. Moreover, recent studies have shown that *HSH2D* can be involved in resistance to methotrexate (Methotrexate) [55] and cisplatin [56]. Based on these findings and our data, we speculate that the poor survival prognosis associated with *HSH2D* expression can be a result of successful remodeling of the gene expression landscape, possibly through the action of U-ISGF3 in cells surviving chemotherapy.

### Implications for therapeutic strategies

The upregulation of U-ISGF3-related genes in DOX-surviving proliferative cells suggests that targeting the action of the U-ISGF3 complex could be a potential strategy to overcome resistance. However, it is not known if JAK/STAT inhibition carries any potential risks. For example, although activation of STAT3 and STAT5 is a poor prognostic factor in many cancers, in other types, it can be favorable [57]. Nevertheless, in many studies, including clinical trials, the main targets of the JAK/STAT pathway are JAKs, whose activity is blocked by inhibitors leading to decreased STAT phosphorylation [57].

For instance, a recent clinical trial with the JAK inhibitor ruxolitinib showed a lack of clinical benefit in patients with triple-negative inflammatory breast cancer [58]. On the other hand, ruxolitinib had antitumor effects in a preclinical model of head and neck squamous cell carcinoma [59]. Nevertheless, in addition to JAK inhibitors that target phosphorylated STATs, directly targeting the U-ISGF3 complex may be more beneficial, as our data and others [16,35] suggest the involvement of the unphosphorylated forms of STAT1/STAT2 in resistance. IRDS genes leading to chemoresistance could be attractive potential targets for future therapies, possibly through modulation of their transcriptional bursting dynamics to reduce the expression of resistance-related genes.

In conclusion, our study provides a comprehensive view of the transcriptomic profiles and alterations in gene co-expression patterns in DOX-treated colon cancer cells. By elucidating the signature of the U-ISGF3 complex and its potential impact on transcriptional bursting of known downstream genes, we identify potential targets for designing therapeutic interventions. Our findings emphasize the need for novel strategies that go beyond inhibiting phosphorylated STATs to address the challenges of chemoresistance effectively. Further research into the mechanisms regulating U-ISGF3 activity and its downstream effects could pave the way for innovative treatments aimed at improving the efficacy of chemotherapy in colorectal cancer.

### Limitations of the study

Although DOX leads to formation of polyploid senescent HCT-116 cells, other cell types can become senescent without polyploidy, which means that our results cannot be generalized to all cell and senescence types. This also has other implications. We cannot identify polyploid cells based on scRNA-Seq. Therefore, we classify DOX-surviving proliferative cells and senescent cells based on scRNA-Seq clustering and commonly used markers of proliferation and senescence. For bursting analysis, we assumed that DOX-surviving proliferative cells are diploid, and we cannot entirely rule out the possibility that some of these cells might be polyploid. We accounted for this limitation by fitting the model assuming polyploidy and found that this does not change the interpretation of our data, indicating that regardless of the number of alleles, U-ISGF3-related genes increase expression by modulation of their burst size. Another assumption we made in modeling was that mRNA decay is not affected by DOX. Since the frequency of bursting, but not burst size, correlates with mRNA decay, this assumption should not change the observed result on burst size.

## Methods

### Cell culture

HCT-116 cells, obtained from the American Type Culture Collection (ATCC), were cultured in McCoy's 5A medium supplemented with L-Glutamine (Gibco), 10 % fetal bovine serum (Gemini), 100 U/ml penicillin and 100 *µ*g/ml streptomycin (Gibco) at 37 °C with 5 % CO_2_.

### Doxorubicin treatment

HCT-116 cells were treated with 200 nM doxorubicin (Sigma) for 24 hours. Following the 24-hour treatment, the doxorubicin-containing medium was removed from the cells. To ensure complete removal of any residual doxorubicin, the cells were washed thoroughly 3x with PBS. The cells were then allowed to recover over a period of 6 days in doxorubicin-free conditions, with the medium being replaced with fresh one, three times during this period. After the 6-day recovery period, the cells were subjected to flow cytometry, microscopy, bulk mRNA sequencing, and scRNA-Seq.

### Flow cytometry

To assess a cell cycle HCT-116 cells were fixed with 4 % paraformaldehyde (Electron Microscopy Sciences) and incubated with 10 µg/mL DAPI (Sigma-Aldrich) in the presence of Triton X-100 (Sigma-Aldrich) for 30 minutes on ice.

Staining of β-galactosidase activity was performed using CellEvent™ Senescence Green Flow Cytometry Assay Kit (Invitrogen) according to the protocol. To gate live cells only, we used LIVE/DEAD™ Fixable Dead Cell Stain (Invitrogen). Cells were analyzed with BD FACSCelesta™ Cell Analyzer.

### Microscopy

The samples were mounted in Prolong Gold with DAPI (Invitrogen), allowed to dry overnight and imaged with Nikon Ti2 microscope, using a 63× oil immersion objective with a pixel size= 111.6 nm.

### Bulk mRNA-Seq

HCT-116 cells (0.5 million) were lysed in 100 µl of lysis solution (RNAqueous™-Micro Total RNA Isolation Kit from Invitrogen). RNA was isolated and treated with DNase following the manufacturer's protocol. High RNA quality was confirmed using the Agilent TapeStation system before proceeding to library preparation. mRNA polyA- purification, reverse transcription and library preparation were conducted using NEBNext Poly(A) mRNA Magnetic Isolation Module, and NEBNext Ultra II Directional RNA Library Prep Kit for Illumina (New England Biolabs). mRNA-Seq was performed in three replicates.

### Bulk mRNA-Seq analysis

Reads were aligned to the human genome (hg19) using gsnap, without detecting splice junctions de novo (–novelsplicing=0) [60]. Existing splice junctions from the RefSeq annotation were taken into account (–use-splicing=/path/to/mm9.splices.iit). The output files were filtered to exclude unaligned reads and alignments with a mapping quality below 20. Reads were then mapped to RefSeq genes using “htseq-count” with the “-m intersection-nonempty” option; basemean, fold-change (FC) and adjusted *p*-values for the differentially expressed gene analysis were calculated using the R package DESeq2 [61]. Genes with >100 basemean, greater than 1.5 FC and <0.001 adjusted p-values were selected as the significantly differentially expressed genes. For pathway enrichment analysis, the ReactomePA R package was utilized [62].

### U-ISGF3-induced genes

A list of U-ISGF3-induced genes was obtained from Cheon et al. [16].

### scRNA-Seq

scRNA-Seq was performed using 10x Genomics system. Libraries were prepared following the manufacturer's protocol for Chromium Single Cell 3′ Reagent Kits (10x Genomics). scRNA-Seq was performed in two replicates.

### scRNA-Seq analysis

We utilized CellRanger (10x Genomics) pipeline (v7.2.0) and bcl2fastq (Illumina) (v2.20) for generating and aligning fastq files to the human genome (hg19), and Seurat R package (https://satijalab.org/seurat/) for further analysis [63]. For DOX-treated samples, we used –force-cells 8000 options for CellRanger count function to include cells which responded to the drug and have smaller number of expressed genes than untreated cells. We applied filtering to remove low-quality and dying cells. To do that, we only analyzed cells with nFeature_RNA < 12000, nCount_RNA < 150000 and percent.mt (the percentage of reads that map to the mitochondrial genome) < 8 %. In the next step, we removed genes that were expressed in <10 cells. Clustering was performed using the Harmony function: samples were integrated, aligned, and clustered using the Harmony method, selecting the first 30 principal components from the PCA linear dimensional reduction of the scaled data. We applied a resolution cutoff of 0.1 for clustering, which resulted in well-segregated clusters 1 and 2.

### Model used to infer bursting kinetics

To fit the two-state telegraph model, we used a Julia software package StochasticGene.jl (v0.7.8.) (https://github.com/nih-niddk-mbs/StochasticGene.jl). The posterior distributions for the rate parameters were estimated using a Bayesian Metropolis-Hastings MCMC algorithm. To obtain rates in a timescale (minutes), we used genome-wide measured mRNA half-lives in HCT-116 cells [19]. To determine the number of alleles in HCT-116 cells we used Hi-C data [19]. To remove low quality rates estimates we performed quality control filtering; (k*_on_* MAD / k*_on_* Median) < 0.75), (Burst Size MAD / Burst Size Median) < 0.75 and Expression > 0.01. Bursting analysis was performed in untreated and DOX-treated cells from cluster 1. All the rates can be found in Table S5.

### Gene co-expression analysis

To compute *Pearson* correlation coefficients between genes pairs across single cells we utilized scRNA-Seq normalized counts matrix, with genes as rows and single cells as columns. The log-normalized data was converted to a matrix format using data.matrix() function. Subsequently, the matrix was transposed using the t() function to prepare for correlation analysis. Finally, *Pearson* correlation coefficients between all gene pairs were calculated using the cor() function, resulting in a gene-gene correlation matrix (Tables S3-4). To organize genes based on their correlation patterns, we performed hierarchical clustering. Next, we generated a heatmap to visualize the gene-gene correlation matrix using the heatmap() function. The heatmap incorporated the hierarchical clustering dendrograms based on DOX sample. Gene co-expression analysis was performed in untreated and DOX-treated cells from cluster 1.

### Statistical analysis

Statistical analysis was done using Wilcoxon test in R, and details are provided in the Fig. legends.

## Author contributions

PT conceived the project, performed experiments and analyses. SKJ performed the computations and analyzed the data. CCC developed the model and wrote the code for StochasticGene. JOS and CCC supervised the project. PT wrote the first draft of the paper. YK, JOS and CCC edited the paper. All authors reviewed the manuscript.

## Data and code availability

Data underlying this study were deposited to NCBI Gene Expression Omnibus (GEO) (http://www.ncbi.nlm.nih.gov/geo) under number: GSE275330. Julia package StochasticGene can be installed directly from Julia and is also available at: https://github.com/nih-niddk-mbs/StochasticGene.jl.

## Declaration of competing interest

The authors declare that they have no known competing financial interests or personal relationships that could have appeared to influence the work reported in this paper.

## Appendix Supplementary materials

Image, application 1

Table S1. A list of differentially expressed genes upon DOX treatment; based on bulk mRNA-Seq. Table S2. A list of differentially expressed genes involved in IFN signaling upon DOX treatment; based on bulk mRNA-Seq. Table S3. Correlation matrix – DOX-treated cells from cluster 1; based on scRNA-Seq. Table S4. Correlation matrix – untreated cells from cluster 1, based on scRNA-Seq. Table S5. Transcriptional bursting parameters – DOX-treated and untreated cells from cluster 1; based on scRNA-Seq. Image, application 2

Image, application 3

Image, application 4

Image, application 5

Image, application 6
