# Supplementary material for: Doxorubicin resistance involves modulation of interferon signaling, transcriptional bursting, and gene co-expression patterns of U-ISGF3-related genes
Source: Neoplasia. 2024 Oct 13;58:101071. doi: 10.1016/j.neo.2024.101071 (PMC11574522; doi:10.1016/j.neo.2024.101071)
Supplement: Supplementary file 1 [file mmc1.docx]

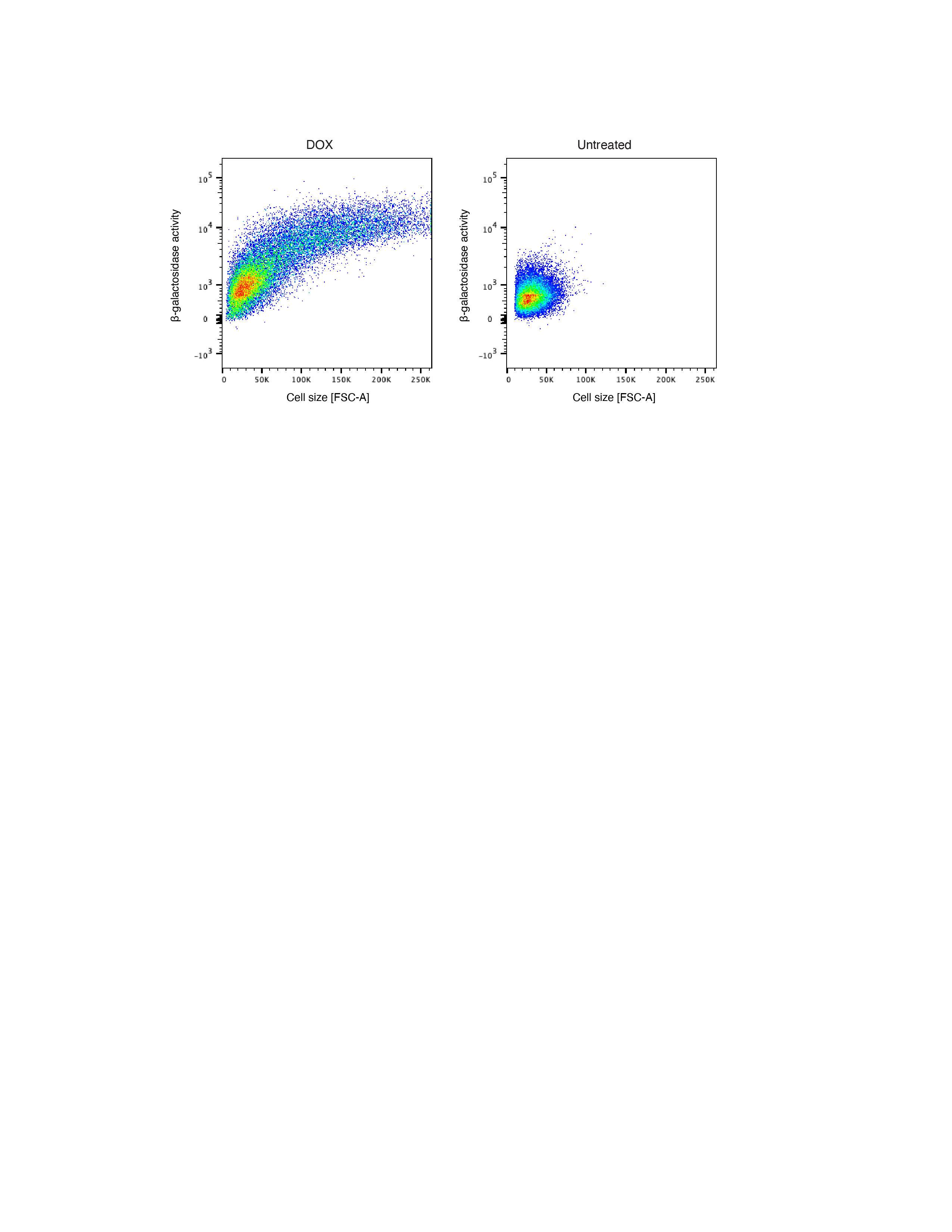


­**Figure S1. The response to doxorubicin treatment varies among cells.**

Flow cytometry panels of DOX (left) and untreated (right) cells presenting staining for the senescence marker: β-galactosidase activity vs. cell size based on forward scatter area (FSC-A).


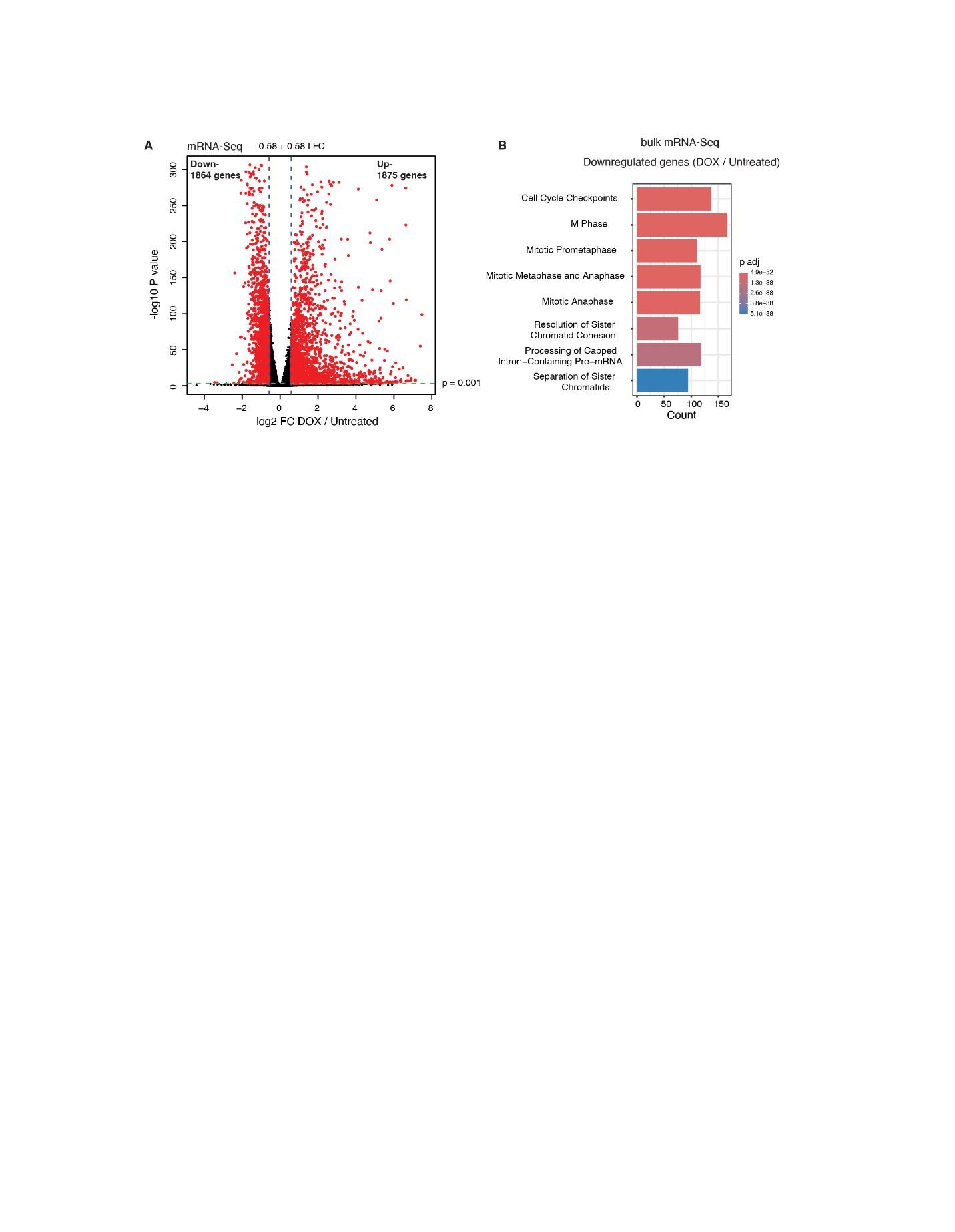


**Figure S2. Doxorubicin treatment affects hundreds of genes.**

(A) Volcano plot presenting down- (n = 1864) and upregulated (n = 1875) genes upon DOX treatment based on bulk mRNA-Seq with a log2 fold change threshold = +/- 0.58. Significantly affected genes are marked in red.

(B) Function of downregulated genes upon DOX treatment as revealed by bulk mRNA-Seq.


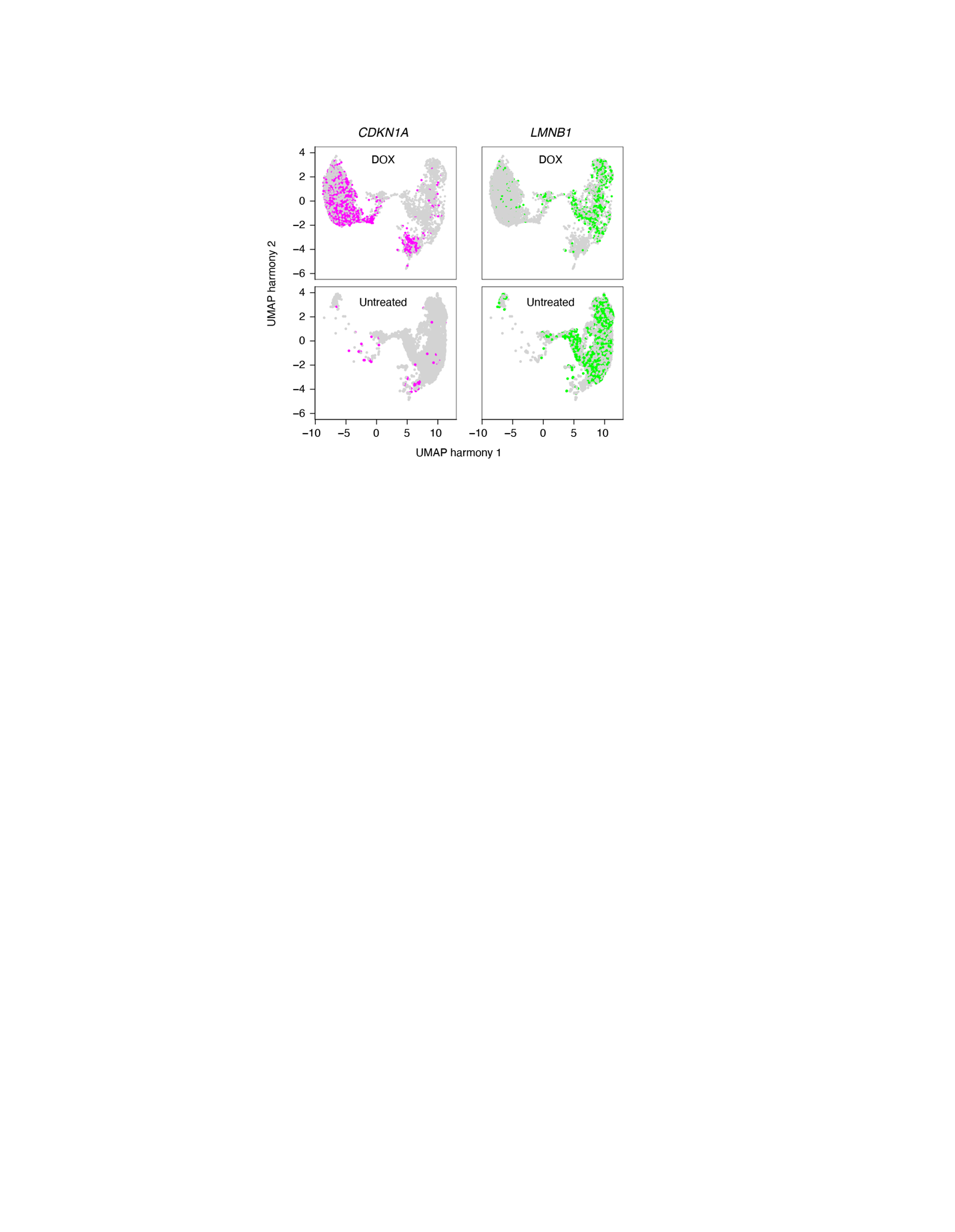


**Figure S3. Impact of doxorubicin on expression of senescence markers across the scRNA-Seq clusters.**

scRNA-Seq UMAPs of doxorubicin-treated (top) and untreated (bottom) HCT-116 cells with indicated cells expressing *CDKN1A* – a gene typically upregulated in senescent cells (in magenta) and *LMNB1* – a gene typically downregulated in senescent cells (in green).

**
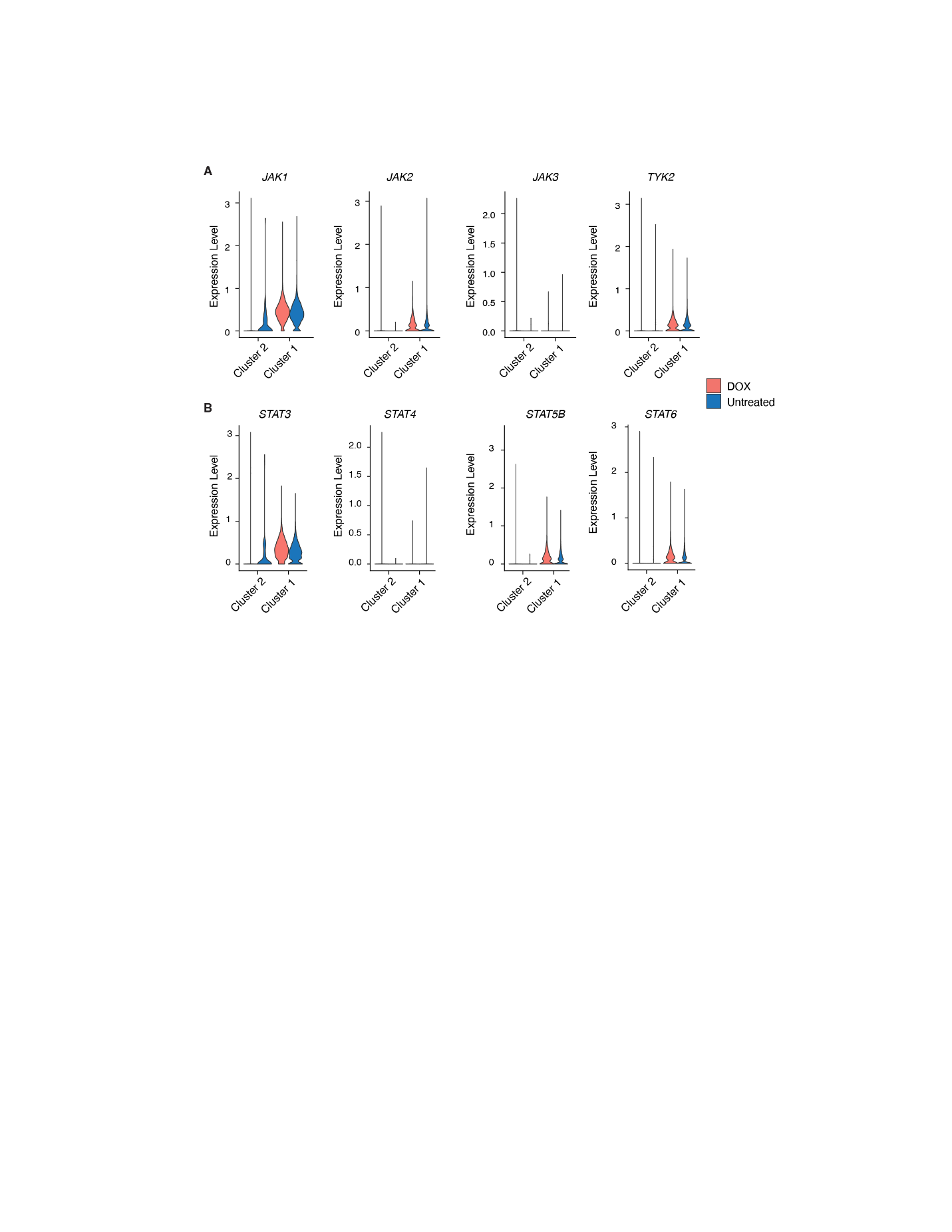
**

**Figure S4. DOX-surviving proliferative cells express JAK/STAT genes.**

(A) Violin plot presenting expression of *JAK* genes in untreated (in blue) and DOX-treated cells (in red). The expression within clusters 2 and 1 is shown.

(B) Violin plot presenting expression of *STAT* genes in untreated (in blue) and DOX-treated cells (in red). The expression within clusters 2 and 1 is shown.


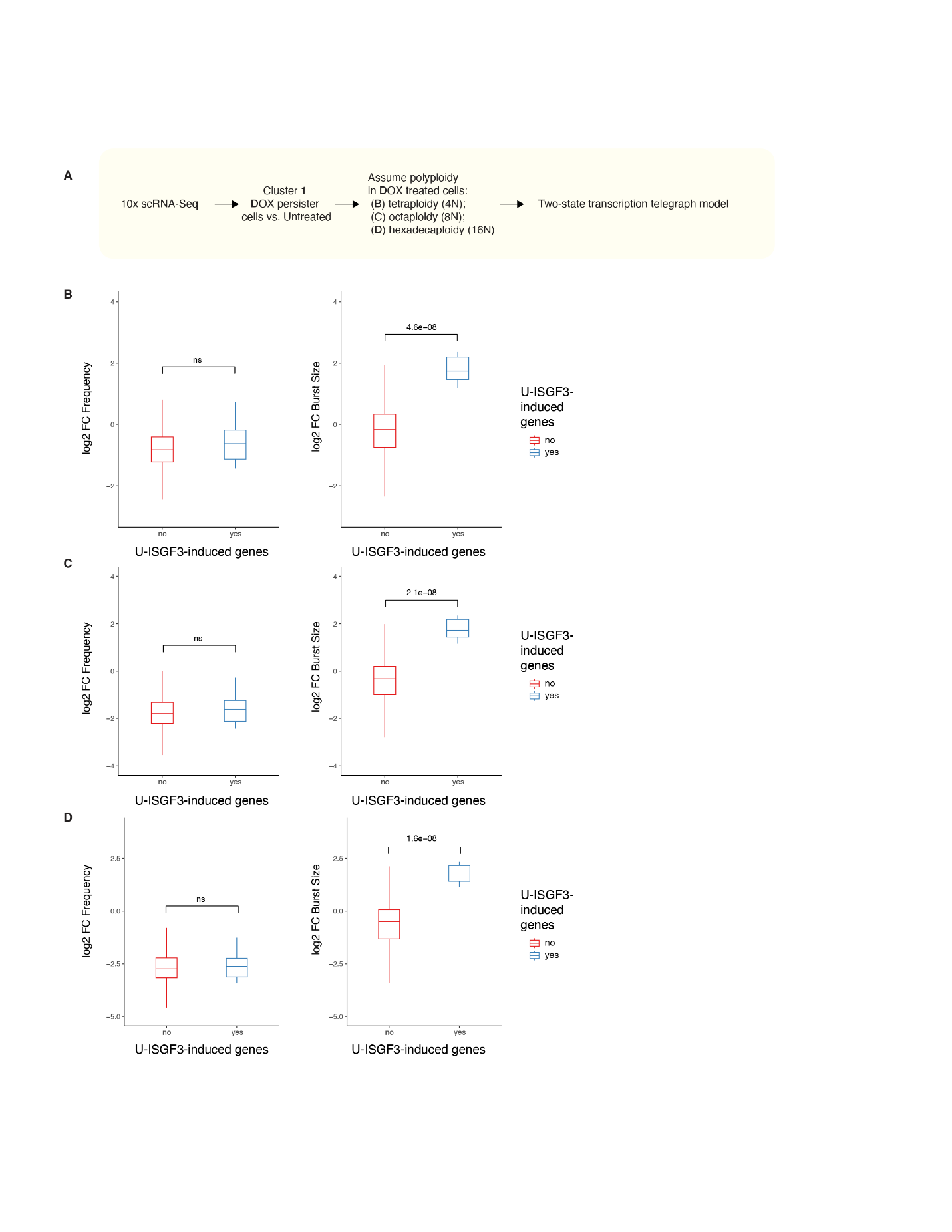


**Figure S5. Regardless of polyploidy, U-ISGF3-related genes are transcribed with a larger burst size.**

(A) scRNA-Seq was performed on untreated and DOX-treated HCT-116 cells. scRNA-Seq histograms of mRNA per cell were obtained from cells within cluster 1, and the two-state telegraph transcription model was fitted to histograms for each gene. As a control, to ensure that the number of alleles does not change the interpretation of the results in Fig. 4C, we refit the data assuming polyploidy of DOX-treated cells from 4 copies of each chromosome (tetraploidy) through 8 (octaploidy) to 16 copies (hexadecaploidy).

(B) Assuming tetraploidy: Box plots presenting log2 fold change (DOX/Untreated) of frequency (left) and burst size (right). In blue: U-ISGF3-induced genes^16^, and in red, all other genes. P-values are shown from the Wilcoxon test, n_yes_ = 12 and n_no_ = 6173 genes.

(C) Assuming octaploidy: Box plots presenting log2 fold change (DOX/Untreated) of frequency (left) and burst size (right). In blue: U-ISGF3-induced genes^16^, and in red, all other genes. ns: not significant; the Wilcoxon test, n_yes_ = 12 and n_no_ = 6423 genes.

(D) Assuming hexadecaploidy: Box plots presenting log2 fold change (DOX/Untreated) of frequency (left) and burst size (right). In blue: U-ISGF3-induced genes^16^, and in red, all other genes. ns: not significant; the Wilcoxon test, n_yes_ = 12 and n_no_ = 6869 genes.

Only the genes that passed the pre-analysis quality control filter have been presented here.
